# Improving emotional health and self-esteem of Malaysian adolescents living in orphanages through Life Skills Education program: A multi-centre randomized control trial

**DOI:** 10.1371/journal.pone.0226333

**Published:** 2019-12-26

**Authors:** Marjan Mohammadzadeh, Hamidin Awang, Suriani Ismail, Hayati Kadir Shahar

**Affiliations:** 1 Department of Community Health, Faculty of Medicine and Health sciences, Universiti Putra Malaysia, Serdang, Malaysia; 2 Department of Psychiatry, Faculty of Medicine and Health sciences, Universiti Putra Malaysia, Serdang, Malaysia; Temple University, UNITED STATES

## Abstract

Generally speaking, institutionalised children and adolescents are at greatly increased risk of serious mental and behavioural problems, up to seven times more than their peers. Life skills- based interventions using peer enforcement have been introduced as effective program to improve adolescents’ emotional and behavioral health. Therefore, the current randomized control study aimed to determine, if a life skills-based intervention could improve the emotional health and self-esteem among Malaysian adolescents in orphanages. Overall, 271 male and female adolescents (13–18 years old) from 8 orphanages in Klang valley, Malaysia participated in the study. Comparing the intervention to control group within 3 points of time, the finding of the study showed that immediately after finishing the interventional sessions (post-test), the mean scores of depression (F = 33.80, P<0.001, η^2^ = 0.11), anxiety (F = 6.28, P = 0.01, η^2^ = 0.02), stress (F = 32.05, P<0.001, η^2^ = 0.11) and self-esteem (F = 54.68, P<0.001, η^2^ = 0.17) were significantly decreased compared to the pre-test values. However, there was no significant difference between two groups in the depression mean scores (F = 2.33, P = 0.13). Regarding to the interaction between group and test a significant change was seen in the mean score of all 4 variables including depression (F = 31.04, P<0.001, η^2^ = 0.10), anxiety (F = 14.21, P<0.001, η^2^ = 0.05), stress (F = 15.67, P<0.001, η^2^ = 0.06) and self-esteem (F = 13.29, P<0.001, η^2^ = 0.05). Furthermore, except depression (Δmean = -1.37, P<0.001), no significant difference was seen between study variables’ mean scores between post- and follow-up test (p>0.001). These results provide preliminary approve for LSE to enhance emotional health and self-esteem in orphanages.

## Introduction

Social and emotional well-being is the departure of childhood and adolescent [1, 2] health development [3, 4] and irreplaceable assets to asses children and adolescents go through any barrier that might happen, thrive in the face of challenging circumstances, avoid risky behavior and generally live a productive life [5, 6]. However, many children and adolescents, even in developed countries, face different levels of mental and emotional difficulties, serious enough to interfere with their development and impair their functions [7, 8].

Even adolescents are normally perceived as a healthy age group, yet, 20% of them in any given year experience a mental health problem, mostly depression and anxiety [9]. In some cases, emotional problems in adolescence continue into the adulthood, causing several problems and harms to individuals and even communities [10].

On the other hand, worldwide, an estimated 153 million children and adolescents, between newborns and 18 years old, have lost one or both of their parents, and daily, 5760 more children lose at least one parent. It is projected that there will be around 500 million orphaned children all around the world by the end of 2018[11].

Starting of the current century, the number of institutionalised children and adolescents has increased rapidly. As adoption is not yet available or easygoing process in many countries, specially developing and undeveloped ones, institutional care such as orphanages are still one of the main options or even the only one for those who are not fortunate enough to have parents with whom to live [12]. It is estimated that more than 70% of children and adolescents living in institutions have at least one living parent living in institutions because their parent(s) are not able to look after them [13]. In many cases, the care and support providing by orphanages focus on basic biological needs such as nutrition, very primary health care and basic education and not mental, emotional and behavioural health issues.

Children and adolescents who live in orphanages are at greatly increased risk of serious mental and behavioural problems [14, 15] such as depression, anxiety, personality problems, coping and adjustment problems as well as low self-esteem [16], up to seven times more than their peers [17]. More than 80% of children and adolescents in orphanages and foster care homes have significant mental health problems, while the prevalence for adolescents in the general population is approximately 20% [13].

In Malaysia in 2010, more than half a million children lost one or both parents due to any cause. As adoption is very long complicated process in Malaysia, orphanages are still the most common method of placement for orphans compared to fostering and adoption [18].

The majority of orphanages and children residential homes in Malaysia are not registered to any formal organization, and there are no reliable statistics of children and adolescents residing in them [19]. However, at least 15,000 children and adolescents are living in almost 90 private and 35 government-run registered institutions and many more in non-registered ones throughout Malaysia. More than 80% of these children and adolescents have at least one living parent. Absence of even a reliable statistics of the number of orphanages and their residents [13] shows the institutional children and adolescents in Malaysia mostly are under-supported, underserved and vulnerable.

Only a few studies have investigated mental and behavioural well-being among children and adolescents in Malaysian orphanages. Therefore, information in this area is very limited. However, results of a study in 2018 showed compared with adolescents living with their families, the prevalence of emotional problems among the adolescents living in residential foster care homes was significantly higher [20]. As well, a study in 2017 by Mohammadzadeh et al, revealed a very high prevalence of depression (85.2%), anxiety (79.4%), stress (86.1%) and low self-esteem among the Malaysian orphanage residences [21]. Results of a local study of three orphanages in Kelantan reported alarming figures of 4%, 19% and 28% of orphans who reported severe, moderate and mild levels of depression, respectively, indicating a high percentage of depression among institutionalised adolescents in Malaysia [22]. Another study in 2015 showed more than 10% of the Malaysian adolescents living in the selected residential homes suffered from Major Depression Disorder (MDD)[23]. In 2017, the results of a study by Mohammadzadeh et al. revealed that almost 80% of adolescents in the selected orphanages in Malaysia suffered from a level of depression, anxiety or stress [21]. Furthermore, according to Women, Family and Community Development Ministry’s report in 2015, almost all of children and adolescents in Malaysian orphanages had low self-esteem and self-confidence [24]. Although much more studies and information needed to figure the exact situation of emotional problems in Malaysian orphanage, this information is likely to be enough to sound an alarm in orphanages in Malaysia.

According to the World Health Organization (WHO), life skills are “*abilities for adaptive behaviour that enable individuals to deal effectively with the demands and challenges of everyday life*” [25]. Deficiency of life skills is conducive to psychological difficulties. Life skills education (LSE) is a structured evidence-based plan that aimed to enhance psychological health and positive and adaptive behaviors among different groups in community[26].

Malaysian adolescents were placed at bigger risk of psychological and behavioural problems such as depression, low self-esteem and bullying due to the lack of appropriate coping and life skills to face with the unexpected adolescence-related challenges [27]. Undoubtedly, the importance of life skills for vulnerable adolescents, including orphanages’ residences, is much higher than that of their average peers [28]. Institutionalised children and adolescents are one of the parts of society which will be future adults and parents; therefore, paying special attention to their mental and behavioural health could have positive effects on general public health in each society.

### The current study

Adolescence is a risky stage for emotion concerns, and has been signalized as a vital period for intervention [29]. However, reviewing literatures reflects a significant lack of systematic LSE program among Malaysian adolescents, specifically in the orphanages. Life skills based interventions using peer enforcement have been introduced as effective program to improve adolescents’ emotional and behavioral health. Taking these considerations into account and as the first randomized controlled trial in Malaysian orphanages, the current study amid to evaluate the effects of a life skills-based intervention programme on emotional health and self-esteem of adolescents in Malaysian orphanages.

The study had 2 main research hypotheses: 1) there is a significant difference in emotional problems mean scores between groups (intervention and control) and within 3 point of time (pre-, post and 4 month follow-up tests); 2) There is a significant difference in self-esteem mean score between groups (intervention and control) and within 3 point of time (pre-, post and 4 month follow-up tests). Regarding to the study objectives, the conceptual framework of the current study explains the foundation of the devolvement of LSE program stemming from Stress-Coping Theory by Lazarus in 1966 [29].

## Methods and materials

### Participants and procedures

**Fig 1:**
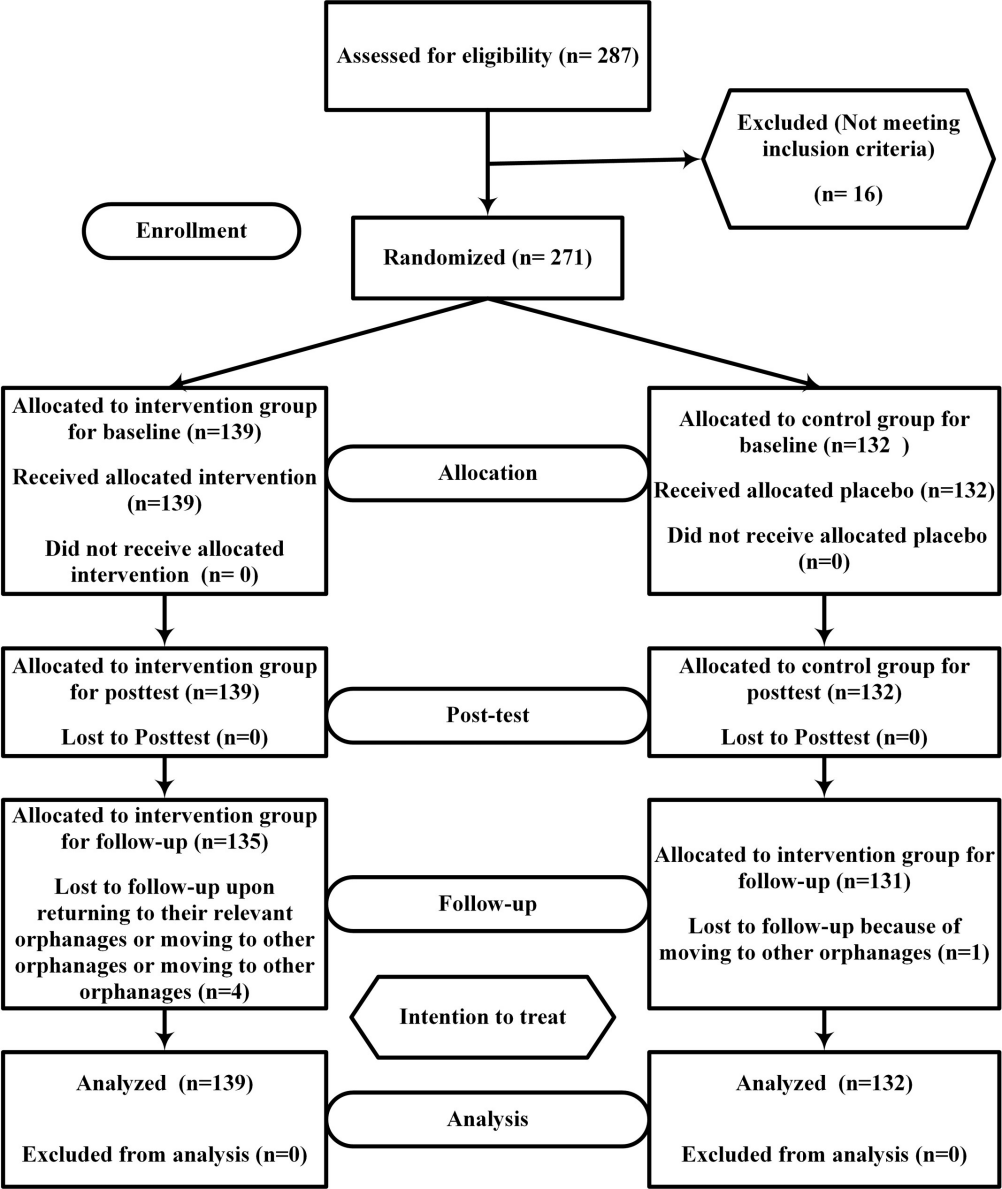
A flow diagram of the study based on the CONSORT2010 statement.

This study was a parallel single-blind (subject-masked) randomized controlled trial (RCT) (S1 File). For this study, the participants were randomly divided into intervention and the placebo control groups (Fig 1). At the time of finalizing the research proposal, clinical trial registration was not needed for educational clinical studies in Universiti Putra Malaysia. However, the study was registered in Thai Clinical Trials Registry in 2014 (Thai Clinical Trials Registry: TCTR20161010003) to fulfill international guidelines. It is confirmed that the related factors for this educational intervention were registered completely and this protocol is the version that submitted and approved by the ethical committee of the Universiti Putra Malaysia before the trial began [Reff.: UPM/TNCPI/RMC/1.4.18.1 (JKEUPM)/F2; 10^th^ Jan 2014]. More details of the study method could also be found in our previous paper [4].

The sample population in the study included all adolescents in the selected orphanages meeting the study’s inclusion and exclusion criteria:

- Inclusion:
  1. Having scores of at least mild in one or more sub-scales of the DASS-21 (depression, anxiety, and depression), based on the results of the screening study;
  2. Aged between 12 and 18 years old;
  3. Having age-appropriate ability to read the study questionnaire and answer to them (Malay Language/ Bahasa Melayu).
- Exclusion:

1. Diagnosed with or treated for psychiatric illnesses (based on their written profile and health condition file records available in the home);
2. Having physical disabilities which prevent their participation in study-related activities, such as blindness and deafness.

### Sample size determination

**Fig 2:**
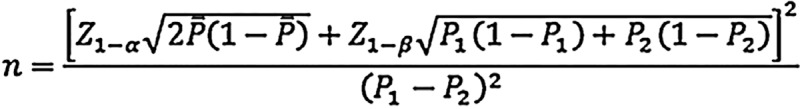
Sample size formula.

Regarding the sample size, to test the difference in proportions between two samples, the equation (Fig 2) by Lwanga and Lemeshow (1991) was used as the sampling formula [30].

In this equation: n = Sample size for each group; Z = Confidence level at 95%; Z_1-a_ = 1.96; Z_1-b_ = 0.84; Power = 80%; Z _a_ = Z_1-a/2_ = 1.28; Z _b_ = Z_1-b_ = 0.84; P_1_ and P_2_ = the proportion of variable before and after intervention in previous studies; $\bar{P}$ = (P_1_ + P_2_) /2. The calculated sample size after adding 20% to enhance the external validity was 128 for each group. The full details of calculation the study sample size is described in our previous publication [4].

### Sampling technique

Using a multi-stage sampling technique, the sampling of the current study was done in 5 steps:

1. Preparing a list of active orphanages in the study location in 2014–2015: regarding to a large number of unregistered orphanages in Malaysia, technically, there is no access to a complete list of these homes; therefore, searches of available databases and resources, such as social media sites and NGOs, were performed to have an appropriate list of homes. The final list included 48 homes.
2. Random selection to select homes for the study: overall, 8 of the 48 identified homes were selected randomly for the study using Microsoft Excel software. The number of homes (8 homes) was selected based on their average population and the calculated sample size.
3. Assigning homes in 2 groups (randomizing): for each of the intervention and control groups 4 homes were selected using simple random sampling.
4. Screening study: all residents of the selected homes, aged 12–18 years, filled the screening questionnaire booklet (287 male and female adolescents) including the socio-demographic and DASS-21 questionnaires.
5. Of the 287 respondents in the screening study, 271 adolescents (136 and 132 adolescents in the intervention and control groups, respectively) scored at least in the mild range in one of the main categories of the DASS-21 questionnaire (depression, anxiety and/or stress) and met the exclusion and inclusion criteria.

### The intervention program

The educational module was developed regarding to the LSE program introduced by World Health Organization (WHO) [31] through a process of consultation with experts in the study field and according to WHO and UNICEF recommendations for teaching life skills. The module was presented in the form of a “guidelines for the training of trainers” booklet (S2 File).

The study’s conceptual framework explains the foundation of the development of the LSE program, which stemmed from the stress-coping theory by Lazarus in 1966 [29]. Study objectives, specific requirements of the target population, the environment in which the participants live, the local culture, ethnic and religious differences and similarities, the special mental health situation of participants and time limitations were some of the important considerations during the development, adoption and design of the activities. To assess the content validity of the interventional module, the initial version of the module was reviewed by 9 experts in child and adolescent psychiatry and psychology and child and adolescent education. The item-level content validity index (I-CVI) and the content validity index for scales (S-CVI) were utilized to calculate the content validity of the interventional module [32]. The minimum **I-CVI** and the computed **S-CVI** for the study module were 0.78 and 0.93, respectively.

Finally, 20 activities were developed and/or rewritten for intervention sessions. The final version of the activities was presented for a panel of expert in Malay to check the validity. The first session included introducing the program and the benefits of learning life skills, discussing the study procedures and educational sessions, answering questions and setting ground rules for the sessions (Table 1). Intervention sessions were held approximately twice weekly for each home for two to two and a half hours per session in Malay language; these sessions included 2 activities as well as about 20-minute break and brief refreshments. Before each activity, the purpose, details and steps of the activity were fully explained for participants. Intervention sessions took various forms, such as role playing, performing drama, drawing, playing games and matches, and having question-and-answer sessions, as well as holding group discussions.

**Table 1:** Content of the interventional sessions [4].

Session	Content	Life Skills	Target(s)
1	**Introduction and icebreaker (one and a half hours):** Introducing life skills, benefits and process of program; establishing the ground rules for program; introducing the research team and preparing the list of participant names; reading and signing the informed consent; Questions and answers. **Main activity:** Activity 1: I am Aware of Myself!	Self-awareness, critical and creative thinking	Self-esteem, emotional health
2	**Short discussion about the last session** **Main activities:** Activity 2: I Want to Learn Life Skills, Let’s Start! Activity 3: I Can Manage My Relationships!	Self-awareness, critical and creative thinking, communication	Self-esteem, emotional health
3	**Short discussion about the last session** **Main activities:** Activity 4: My Friends Are the Most Important Part of My Life! Activity 5:I Understand the Importance of Proper Communication!	Self-awareness, critical and creative thinking, communication and intra-and interpersonal relationship	Self-esteem, emotional health
4	**Short discussion about the last session** **Main activities:** Activity 6: I Have the Ability to Negotiate Effectively and Say NO! Activity 7:I Can Identify the Problems and Risks!	Self-awareness, critical and creative thinking, communication and problem solving	Self-esteem, emotional health
5	**Short discussion about the last session** **Main activities:** Activity 8: I Make Smart Decisions (1) Activity 9: I Make Smart Decisions (2)	Problem solving, decision making, and critical and creative thinking	Self-esteem, emotional health
6	**Short discussion about the last session** **Main activities:** Activity 10: Stay Calm and React Intelligently (1) Activity 11: Stay Calm and React Intelligently (2)	Self-awareness, problem solving, decision making, critical and creative thinking and communication	Self-esteem, emotional health
7	**Short discussion about the last session** **Main activities:** Activity 12: Stay Calm and React Intelligently (3) Activity 13: Think Again and Find a Better Way (1)	Critical and creative thinking, empathy and coping	Self-esteem, emotional health and coping mechanisms
8	**Short discussion about the last session** **Main activities:** Activity 14:Think Again and Find a Better Way (2) Activity 15:Think Again and Find a Better Way (3)	Coping with emotion and stress, critical and creative thinking and problem solving	Coping mechanisms, self-esteem and emotional problems
9	**Short discussion about the last session** **Main activities:** Activity 16: Stay Calm and React Intelligently (4) Activity 17: Think Again and Find a Better Way (4)	Coping with emotion and stress, critical and creative thinking and problem solving	Coping mechanisms, self-esteem and stress
10	**Short discussion about the last session** **Main activities:** Activity 18:I am Aware of Myself! Activity 19: I am Stronger Now(1)	Coping with emotion and stress, communication and empathy	Coping mechanisms and self-esteem
11	**Short discussion about the last session** **Main activities:** Activity 20: I am Stronger Now(1) **Watching short videos**	Coping with emotion and stress, communication and relationship	Coping mechanisms
12	Final discussion, overview, questions and answers, fun activities.	-------	--------

### Control group

Participants in the control group received 6 sessions (each approximately 45 minutes) over more than a month and a half of the Communication for Behavioral Impact (COMBI) program for preventing and controlling dengue. This program is also part of the WHO “*behavioural-focused social mobilization and communication programmes*” for communicable disease prevention and control in Malaysia [33]. The pre-test, post-test and follow-up tests were performed for the control group, which was the same as the intervention group. Each participant received small gifts before performing the pre-test and follow-up test. Furthermore, all participants were planned to receive the educational program after finishing the follow-up test by the research assistants.

### Study instruments

1. **Demographic Questionnaire:** The demographic questionnaire included some questions about the socio-demographic characteristics of participants: age, gender, race, educational level, parental status and the duration of living in a home(s).
2. **Depression Anxiety Stress Scales (DASS-21):** Designing by Lovibond and Lovibond (1995), The DASS-21 measures negative affect (depression, anxiety and stress) of respondents [34]. The triple sub-scales of this questionnaire includes seven 4-point Likert scale statements which higher scores indicate lower levels of the emotional health factor. For this study, the validated self-administered Malay version of the instrument [35] was used. As well, with regard to the results of the pilot study, the Cronbach’s alpha coefficients for depression, anxiety and stress were 0.81, 0.79 and 0.81, respectively.
3. **Rosenberg Self-Esteem Scale (RSES):** The validated self-administered Malay version of the RSES [36] was utilized as the other instrument in this study to assess the participants’ self-esteem. The RSES includes ten five-point Likert scale statements from 1 to 5 (strongly agree, agree, no idea, disagree and strongly disagree, respectively). Negative statements (2, 5, 6, 8 and 9) should be revised prior to data analysis. For this study, the scale range was from 10 to 50 and scores below 30 identified as low self-esteem [37]. Some studies use 4- or 7-point Likert scales. Scale ranges vary based on the addition of "middle" categories of agreement. In the pilot study, the Cronbach’s alpha coefficients were 0.73.

### Data collection

Prior to the pre-test, the questionnaire statements were explained to participants. The same questionnaire booklet was used for post-test and a 4-month follow-up test. The pre-test and post-test were performed at the beginning of the first session and after the last activity in the last session. The follow-up test was performed 4 months after the post-test (±one week) after a brief review of the training program (Fig 1).

### Ethical consideration

The study was approved by the Ministry of Welfare of Malaysia, Department of Social Welfare (Ref: JKMM 100/12/5/2:2013/180). Furthermore, an approval letter from the Medical Research Ethics Committee of the Faculty of Medicine and Health Sciences of Universiti Putra Malaysia (Ref: UPM/TNCPI/RMC.1.4.13) was obtained to conduct the study.

Before they began the intervention sessions, all caregivers in the selected homes received a fact sheet and a detailed explanation of the study and ethical issues either during a face-to-face meeting or by phone. Furthermore, in the first session, all participants received information and details about the study and intervention sessions as well as written informed consent. They were also informed about their rights as participants and the researchers’ ethical responsibility. At the end, all the participants, caregivers and parents/guardians were asked to sign the written informed consent form.

### Data analysis

Data analysis was conducted using SPSS software version 21 [38] published by IBM Corp. Before the analysis, the data were double checked with searches for incorrect entries and missing data. In order to estimate odds ratios, the confidence interval (CI) was 95%. As well, the level of significance (P-value) was 0.05 and 0.02 (0.05/3) for adjusted P-value, using Bonferroni adjustment.

A mixed between-within-subjects ANOVA was used to assess the means differences of the scale variables in the intervention and control groups. The requested assumptions such as normality and homogeneity of variance and co- variance were checked before running the tests. Partial eta squared was used as a measure of the effect size. According to Cohen (1988), 0.01, 0.06 and 0.14 represent small, moderate and large effect sizes, respectively [39].

### Missing data

Using the intention-to-treat (ITT) strategy [40], missing data treatment was performed through data assessment to determine the amount and distribution of missing values. Overall, 2.9% of the data were missing. Assessing data bias to check if data were missing randomly, the results of Little's MCAR test [41] showed that the data were considered to be missing at random. As the amount of missing data was very small, values were imputed using the expectation-maximization method with importance resembling using SPSS 21 software [42].

## Results

### Demographic and Socio-demographic Characteristics of the Participants

Table 2 presented the socio-demographic characteristics of the participants. Overall, among 271 adolescents participating in this study, 149 (55%) were male and 122(45%) were female. The mean age of the participants was 14.47±1.37 years.

**Table 2:** Socio-demographic characteristics of participants (n = 271).

Characteristic	Frequency	Percentage	Mean±SD
**Age**			14.47±1.37
**Gender**			
Male	149	55%	
Female	122	45%	
**Race**			
Malay	183	67.5%	
Chinese	13	4.8%	
Indian	59	21.8%	
Other	16	5.9%	
**Educational level**			
Primary School	21	7.7%	
Secondary school	238	87.8%	
Other	12	4.4%	
**Parental Status**			
Lost both parents	20	7.4%	
Lost one parent	133	49.1%	
Not living with parents	118	43.5%	
**Duration of stay in homes**			
Less than 6 months	16	5.9%	
6 to 12 months	29	10.7%	
1 to 2 years	78	28.8%	
More than 2 years	148	54.6%	

Most of the participants were of Malay ethnicity (67.5%), followed by Indian (21.8%). Participants with Chinese ethnicity comprised 4.8% of the sample, and sixteen (5.9%) participants were from other ethnic groups, mostly Orang Asli and Indonesian. The highest educational level of the majority of the participants was secondary school (87.8%). Twelve (4.4%) participants had received informal education in the home to prepare them for formal education based on their age.

Most of the participants had lost at least one of their parents (56.5%), while both parents of 118 (43.5%) participants were alive but not able to take care of them for any reason. More than half of the participants (54.6%) lived in homes for more than 2 years, while only sixteen (5.9%) participants had stayed in homes for less than 6 months. No significant differences were found in the demographic and socio-demographic characteristics of the participants between groups.

### Intervention effects

Table 3 shows the result of ANOVA within- and between-subjects effects for emotional problems (depression, anxiety and stress) and self-esteem. There was a significant difference in the mean scores for depression (F = 33.80, P<0.001, η^2^ = 0.11) among the 3 time points. The mean scores for anxiety (F = 6.28, P = 0.01, η^2^ = 0.02), stress (F = 32.05, P<0.001, η^2^ = 0.11) and self-esteem (F = 54.68, P<0.001, η^2^ = 0.17) were significantly different between groups, but there was no significant difference between the two groups in the mean scores for depression (F = 2.33, P = 0.13).

**Table 3:** Results of ANOVA within- and between-subjects effects for emotional variables and self-esteem.

Variable		Type III Sum of Squares	df	Mean Square	F-Value	P-Value	Partial Eta Squared
**Depression**	Time	151.18	2	75.59	33.80	<0.001*	0.11
	Group	66.76	1	66.76	2.33	0.13	0.01
	Time * Group	138.85	2	69.43	31.04	<0.001*	0.10
**Anxiety**	Time	206.93	1.81	114.33	11.70	<0.001*	0.04
	Group	103.90	1	103.90	6.28	0.01*	0.02
	Time * Group	251.27	1.81	138.82	14.21	<0.001*	0.05
**Stress**	Time	615.81	1.86	330.90	28.13	<0.001*	0.10
	Group	534.95	1	534.95	32.05	<0.001*	0.11
	Time * Group	343.09	1.86	184.36	15.67	<0.001*	0.06
**Self-Esteem**	Time	1371.562	2	685.78	19.03	<0.001*	0.07
	Group	2212.349	1	2212.35	54.68	<0.001*	0.17
	Time * Group	957.717	2	478.86	13.29	<0.001*	0.05

*Significant at p<0.02 level

Regarding the interaction between group and time point, a significant change was observed in the mean score for all 4 variables, including depression (F = 31.04, P<0.001, η^2^ = 0.10), anxiety (F = 14.21, P<0.001, η^2^ = 0.05), stress (F = 15.67, P<0.001, η^2^ = 0.06) and self-esteem (F = 13.29, P<0.001, η^2^ = 0.05), as shown in Table 3.

### Bonferroni test (between groups)

A post hoc test (Bonferroni) was applied to compare the mean scores of variables. The results of the Bonferroni test revealed differences in depression (Δmean = -1.72, P<0.001), anxiety (Δmean = -0.99, p = 0.01), stress (Δmean = -1.97, P<0.001) and self-esteem (Δmean = 5.24, P<0.001) scores between the control and intervention groups at post-test. Furthermore, a significant change was observed in the mean scores for anxiety (Δmean = -1.92, P<0.001), stress (Δmean = -3.01, P<0.001) and self-esteem (Δmean = 4.39, P<0.001) at 4-month follow-up. There was no significant difference between the intervention and control groups at 4-month follow-up (Δmean = -0.18, p = 0.67). The detailed results are presented in Table 4.

**Table 4:** Holistic mean difference between the intervention and control groups at the pre-test, post-test and follow-up test for depression, anxiety, stress and self-esteem.

Variable	Time	Intervention Position (I)	Intervention Position (J)	Mean Difference (I-J)		S.E.	p-value	95% CI		Partial ƞ^2^
								Lower Bound	Upper Bound	
Depression	1	Intervention	Control		0.19	0.38	0.63	-0.57	0.94	0.001
	2	Intervention	Control		-1.72	0.40	<0.001*	-2.52	-0.93	0.06
	3	Intervention	Control		-0.18	0.43	0.67	-1.02	0.66	0.001
Anxiety	1	Intervention	Control		0.76	0.44	0.084	-0.10	1.63	0.01
	2	Intervention	Control		-0.99	0.39	0.012*	-1.76	-0.22	0.02
	3	Intervention	Control		-1.92	0.40	<0.001*	-2.70	-1.13	0.08
Stress	1	Intervention	Control		0.11	0.35	0.74	-0.57	0.80	0.00
	2	Intervention	Control		-1.97	0.46	<0.001*	-2.87	4.41	0.06
	3	Intervention	Control		-3.01	0.49	<0.001*	-3.97	-2.05	0. 12
Self-Esteem	1	Intervention	Control		0.27	0.72	0.71	-1.16	1.70	0.01
	2	Intervention	Control		5.24	0.73	<0.001*	3.80	6.68	0.16
	3	Intervention	Control		4.39	0.78	<0.001*	2.86	5.91	0. 11

*Significant at p<0.02 (adjusted P-value)

### Bonferroni test (within groups)

In addition, to show the efficacy of LSE, the mean scores for the study variables at pre-test, post-test and 4-month follow-up were compared in both the intervention and control groups. The results of the post hoc test (Bonferroni) revealed a significant difference between pre-test and post-test for depression (Δmean = 2.00, P<0.001), anxiety (Δmean = 2.04, P<0.001), stress (Δmean = 2.80, P<0.001) and self-esteem (Δmean = -5.48, P<0.001), with a large effect size and a large effect in the intervention group (Table 5).

**Table 5:** The differences in the mean scores for depression, anxiety, stress and self-esteem between time points in the intervention and control groups.

Variable	Intervention Position	(I) Time	(J) Time	Mean Difference (I-J)	S.E.	p-value	95% CI		Partial ƞ^2^
							Lower Bound	Upper Bound	
Depression	Intervention	Pre-test	Post-test	2.01	0.18	<0.001	1.57	2.45	0.32
		Pre-test	Follow-up	0.63*	0.17	<0.001	0.21	1.05	
		Post-test	Follow-up	-1.37*	0.18	<0.001	-1.81	-0.94	
	Control	Pre-test	Post-test	0.10	0.19	1	-0.35	0.55	0.01
		Pre-test	Follow-up	0.26	0.18	0.42	-0.17	0.70	
		Post-test	Follow-up	0.17	0.19	1	-0.29	0.62	
Anxiety	Intervention	Pre-test	Post-test	2.04*	0.35	<0.001	1.20	2.89	0.13
		Pre-test	Follow-up	2.28*	0.41	<0.001	1.30	3.26	
		Post-test	Follow-up	0.24	0.31	1	-0.50	0.97	
	Control	Pre-test	Post-test	0.29	0.36	1	-0.58	1.15	0.02
		Pre-test	Follow-up	-0.40	0.42	1	-1.40	0.60	
		Post-test	Follow-up	-0.69	0.31	0.09	-1.45	0.07	
Stress	Intervention	Pre-test	Post-test	2.80*	0.36	<0.001	1.94	3.66	0.30
		Pre-test	Follow-up	3.49*	0.38	<0.001	2.57	4.41	
		Post-test	Follow-up	0.69	0.45	0.37	-0.38	1.77	
	Control	Pre-test	Post-test	0.71	0.37	0.16	-0.17	1.59	0.02
		Pre-test	Follow-up	0.36	0.39	1	-0.58	1.31	
		Post-test	Follow-up	-0.35	0.46	1	-1.45	0.75	
Self-esteem	Intervention	Pre-test	Post-test	-5.48*	0.71	<0.001	-7.18	-3.78	0.20
		Pre-test	Follow-up	-4.49*	0.71	<0.001	-6.21	-2.77	
		Post-test	Follow-up	0.99	0.74	0.54	-0.79	2.77	
	Control	Pre-test	Post-test	-0.51	0.72	1	-2.25	1.24	0.01
		Pre-test	Follow-up	-0.37	0.73	1	-2.14	1.39	
		Post-test	Follow-up	0.14	0.76	1	-1.69	1.96	

*Significant at p<0.02 (adjusted P-value)

Furthermore, except for depression (Δmean = -1.37, P<0.001), no significant difference was observed between the mean scores for the study variables between the post-test and follow-up test (p>0.001). Meanwhile, there was no significant difference in the mean scores for the study variables between pre-test and post-test as well as between post-test and 4-monthfollow-up in the control group (p>0.001).

**Fig 3:**
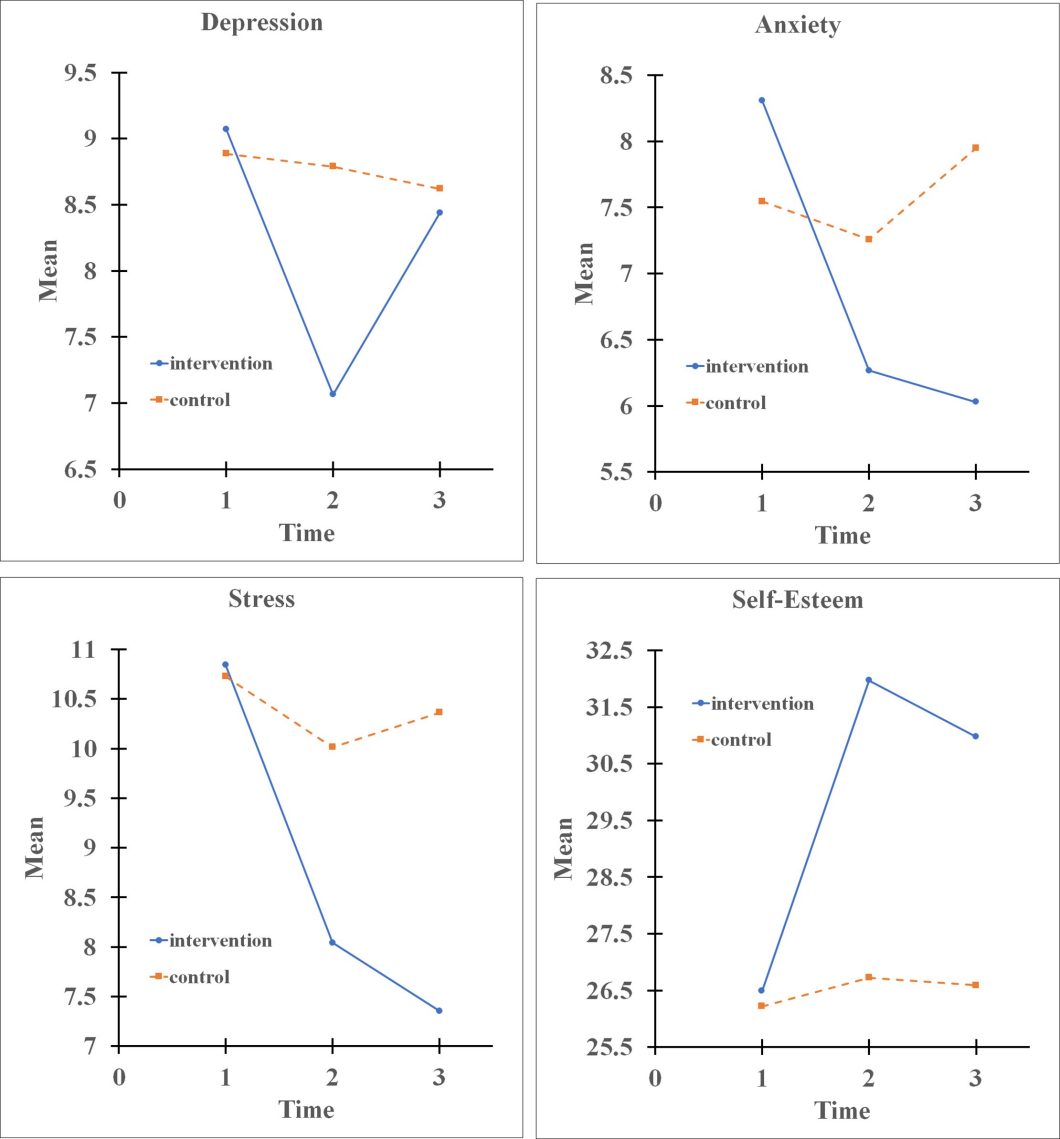
Mean plots for depression, anxiety, stress and self-esteem in the intervention and control groups over time.

A summary of the descriptive statistics of emotional variables and self-esteem scores at 3 different points in time for the intervention and control groups is shown in Table 6. Fig 3 shows the mean plots for depression, anxiety, stress and self-esteem in the intervention and control groups across the 3-stage (pre-test, post-test and 4-month follow-up) tests.

**Table 6:** Descriptive statistics (means, SDs) of the emotional problems and self-esteem scores at 3 different time points for the intervention and control groups.

Variable	TEST	Group	Mean±SD
Depression	Pre-test	Intervention	9.07±3.12
		Control	8.89±3.16
	Post-test	Intervention	7.06±3.20
		Control	8.79±3.43
	Follow-up	Intervention	8.43±3.32
		Control	8.62±3.68
Anxiety	Pre-test	Intervention	8.31±3.56
		Control	7.54±3.69
	Post-test	Intervention	6.27±3.11
		Control	7.26±3.32
	Follow-up	Intervention	6.03±2.88
		Control	7.95±3.66
Stress	Pre-test	Intervention	10.84±2.89
		Control	10.72±2.88
	Post-test	Intervention	8.04±3.36
		Control	10.01±4.12
	Follow-up	Intervention	7.35±3.33
		Control	10.36±4.64
Self-Esteem	Pre-test	Intervention	26.49±5.95
		Control	26.22±5.99
	Post-test	Intervention	31.97±6.04
		Control	26.73±5.98
	Follow-up	Intervention	30.98±7.10
		Control	26.59±5.53

## Discussion

A review of the literature revealed a significant gap in interventional education programs, such as LSE, within residential orphanages in Malaysia. Therefore, to bridge this gap, the current study aimed to evaluate the effects of a life skills-based intervention program on the emotional health and self-esteem of adolescents in Malaysian orphanages. Overall, the results showed that LSE had a significant effect on decreasing the level of emotional problems (anxiety, depression and stress) among participants in the intervention group.

The mean score for depression in the intervention group at pre-test was 9.07±3.12, which decreased to 7.06±3.20 at post-test. On the other hand, the finding of the four-month follow-up revealed a different story. The mean score for depression in the intervention group increased again at the four-month follow-up compared to at post-test (8.43 vs.7.06), although it was still lower than the mean score for depression at pre-test. Therefore, it seems that the intervention program was effective against depression but not enough for a sustainable change after 4 months. This finding may be attributable to the nature of depression, which requires deeper interventions or treatments or even a combination of psychotherapy and pharmacotherapy, especially in severe and very severe cases, to reach a sustainable cure [43]. A review of previous studies examining the effect of LSE on adolescent depression shows that despite evidence of a positive effect of LSE on adolescent depression [44], other studies did not show support for its impact [45]. However, most previous studies had only 2 time points of assessment (pre-test and post-test) and did not follow the effects of their intervention program over time.

Furthermore, the results of the current study showed that LSE had a significant impact on reducing the levels of anxiety and stress among participants in the intervention group. The mean scores for anxiety and stress in the intervention group at pre-test were8.31±3.56 and 10.84±2.89, respectively, which decreased to 6.27±3.11 and 8.04±3.36 at post-test. Additionally, the results of the follow-up test in the intervention group showed the continuity of improvement in the levels of anxiety (6.03±2.88) and stress (7.35±3.33) after 4 months. Therefore, LSE had a more effective sustainable impact on adolescents’ anxiety than on their depression, but among the 3 factors of emotional problems examined in this study, the intervention program had the greatest impact on stress.

Previous studies have confirmed the effect of LSE on the anxiety levels of both institutional and noninstitutional adolescents [46].Additionally, most of the studies investigating the effectiveness of LSE on stress have supported the results of the current study [47, 48].

On the other hand, according to the study’s finding, the mean score for self-esteem in the intervention group at pre-test was 26.49±5.95, which increased to 31.97±6.04 at post-test. Therefore, LSE had a significant impact on improving the levels of self-esteem among participants in the intervention group. Furthermore, the results of the follow-up test in the intervention group showed no significant difference between the mean scores for self-esteem at post-test and at follow-up (30.98±7.10), which showed the sustainability of the effect of LSE on participants’ self-esteem. These results are consistent with those of previous studies assessing the efficacy of LSE-based interventions among adolescents [49], although some of these studies used different instruments, such as Cooper smith’s Self-Esteem Inventory and Pope’s Self-Esteem Questionnaire, to assess the levels of self-esteem among their participants [50, 51].

Therefore, the results of the current study showed that the intervention program can be introduced as an effective plan for improving emotional health and self-esteem among Malaysian adolescents living in orphanages. As with other programs introduced by the WHO and UNICEF, LSE is cost-effective and easy to administer by local trainers without requiring specific tools. Therefore, as a starting point, the findings of the current study could be used by Malaysian educational planners and educational managers to design and implement continuous educational programs based on LSE for institutional and even noninstitutional Malaysian children and adolescents to improve public health in the country.

Additionally, due to the importance and magnitude of the problems of children and adolescents in orphanages, the findings of this research and the educational model in the intervention program are intended to help policymakers, practitioners in the healthcare field, caregivers in orphanages and teachers pay special attention to LSE to promote healthier youth and communities.

Finally, LSE is a new approach in Malaysia and has high potential for study in different populations. Future studies should focus on the effect of LSE on emotional health and self-esteem over a longer time period to assess sustainability. Future studies should also investigate the effects of LSE on other factors of mental and behavioral health among institutionalized and orphaned children and adolescents in Malaysia, such as self-efficacy, antisocial behavior, aggression, and personality issues.

### Study limitations

This study had some limitations. First, the instrument used for the screening study and the main study was the same (DASS 21). Second, the study used self-administered questionnaires. This might cause some bias such as inaccurate data or misunderstanding of the questions. However, providing detailed explanations to the respondents and answering to the questions before data collection minimized this limitation. Next, the time period of the control group educational sessions was shorter than that of the intervention group due to the time limitation. The lack of similar information to compare the current results in Malaysia population was another limitation.

## Supporting information

S1 File CONSORT checklist. (DOC)

S2 File Educational/Interventional protocol (sample). (DOCX)
