# Supplementary material for: Improving emotional health and self-esteem of Malaysian adolescents living in orphanages through Life Skills Education program: A multi-centre randomized control trial
Source: PLoS One. 2019 Dec 26;14(12):e0226333. doi: 10.1371/journal.pone.0226333 (PMC6932807; doi:10.1371/journal.pone.0226333)

**Appendix A**

**Intervention Module (Sample)**


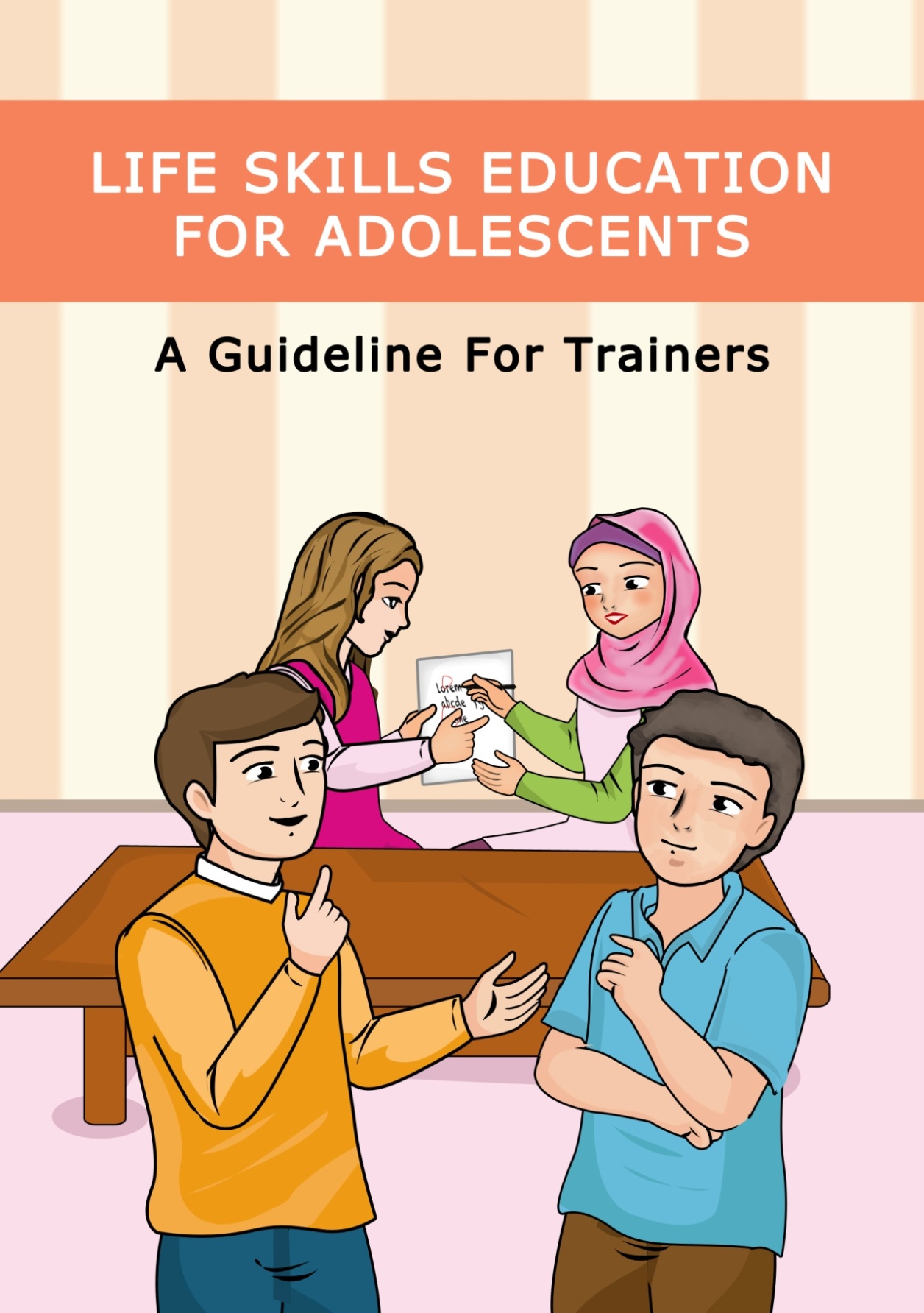


**This booklet is designed to improve life skills and mental health among Malaysian adolescents. To achieving the project objectives, this booklet contains 20 activities.**

**Introduction**

**First thing First!**

Before starting the activities plan, you need to **explain life skills and the importance of learning these skills** clearly and answer all the questions about it. It isn’t time wasting at all if you even spend one whole session to do so. If the adolescents do not have a background and clear understanding of what and why they do the activities, they will not cooperate!

**Do the activities your way based on your situation**

The booklet’s activities can give you an idea and a perspective of how to run the training sessions. You can change all details based on your target age, your time, your goals and your situation.

In fact, it is much better if you use **your words and examples** in the sessions. So get the idea but use your own words which suit your target group.

**Discussion part is the main part**

**Never leave out or underestimate the discussion part** of activities. It helps you finding out what have been learnt in your sessions by your target group and make the session useful. Do your best to make this part attractive and enjoyable.

In this booklet, there are some suggestions at the end of each activity to help you hold the discussion part but you always can use your own words to make it more efficient.

**Ground rules**

At the beginning of the first session do the GROUND RULE activity to make sure all adolescents know about the rules of sessions and agree with them.

**Always use positive reinforcement**

In all sessions, try to have a creative plan to **encourage participants to have better cooperation** in activities. You can give a small gift to the adolescents participating in final discussion actively, preparing some prizes for the winner groups (when there is a competition in activity) and give responsibility to some adolescents and ….

| **Activity 2**  ***I Want to Learn Life Skills, Let’s start!*** |
| --- |
| - **LIFE SKILLES:**   Self awareness, Critical Thinking, Creative Thinking   - **MATERIALS:**   Flash cards, Colourful pens, Blank papers, White board and Markers |
| **Purpose, Details and Important Points:**  This activity is purposed to help adolescents to understand the main factors that they can change to learn and (re)act better in their daily life. Through this activity, adolescents will learn that personality and behaviour are 2 separated factors and bad behaviour is not always related to the bad person. They learn that some other factors such as environment can effect on the behaviours and abilities and in many cases we are able to change our wrong behaviours.  First, adolescents schematically learn about the factors that affect our behaviours including personality, beliefs, abilities and environment. Then, they are divided into some small groups (4 to 6 persons) and under trainer supervision, try to find some factors that they like about themselves and don’t want to change and some others that they can or want to change regarding to their abilities and situations in their real life. Back to the big circle, each team talks about their finding.  Next, participants are asked to think and discuss about: what are the factors that we cannot change in our life? Why? Is failing in some works or situations always our fault? Why yes and why not?  **Final Discussion:** in final discussion, the participants discuss about these questions:   1. What did you learn from the activities today? 2. Was it easy to understand what the session was about? 3. What did you most like/dislike about the session? |

**
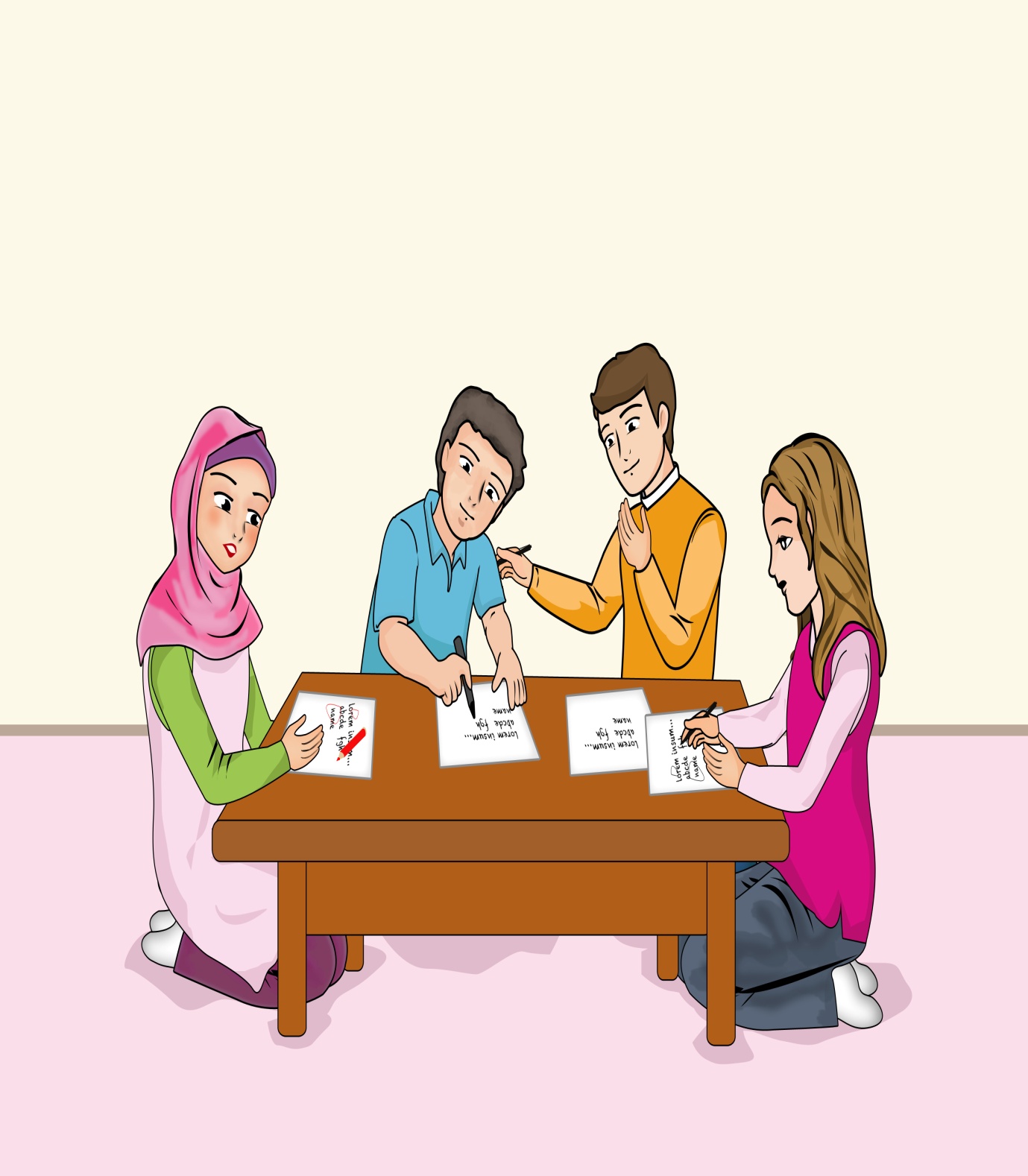
**

| **Activity 3**  ***I Can Manage My Relationships*** |
| --- |
| - **LIFE SKILLES:**   Self awareness, Critical Thinking, Communication, inter- and intra personal relationships   - **MATERIALS:**   Flash cards, Colourful pens |
| **Purpose, Details and Important Points:**  This activity is purposed to help adolescents to understand different kinds of relationships and their importance. Furthermore, through this activity adolescents learn about the basic definition of friend and friendship.  First, adolescents learn about the important groups and relationships in their life including parents, sibling and elders, close relatives (for example aunts and uncles and cousins), friends including boy/girl friends, persons in authority such as teachers and caregivers and other persons around us who are not included in these groups. Then, adolescents are asked to explain the priority and important points in relationship with each group using their own words.  Next, participants are divided into some small groups (4 to 6 persons) and under trainer supervision, try to answer these questions: what are the most important relationships in our life? Why? What are the important points to have a successful relationship? What are the weak points and threats in each group? Gathering in big circler, each group is asked to read their answers and discuss about them with others.  **Final Discussion:** in final discussion, the participants discuss about these questions:   1. What did you learn from the activities today? 2. Was it easy to understand what the session was about? 3. What did you most like/dislike about the session? |

**
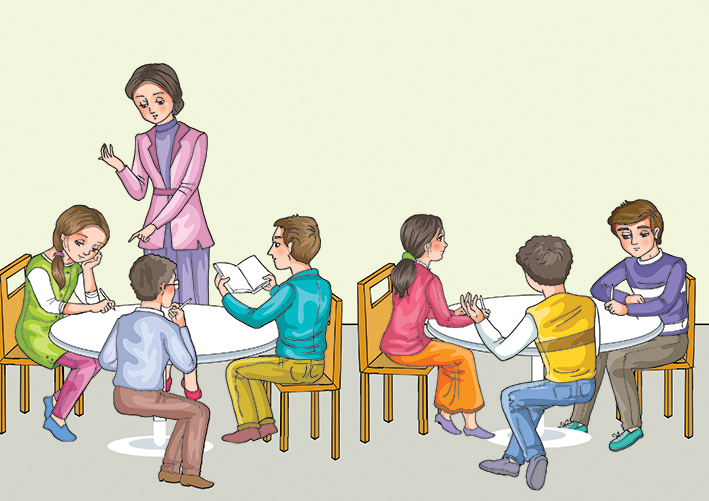
**

| **Activity 5**  ***I Understand the Importance of Proper Communication*** |
| --- |
| - **LIFE SKILLES:**   Critical Thinking, Creative Thinking, Communication, intra-personal relationships   - **MATERIALS:**   Large blank papers, Colorful pens |
| **Purpose, Details and Important Points:**  This activity is purposed to help adolescents to understand the different communication methods and improve their communication and team work skills. Furthermore, this activity helps them to understand the importance of listening well in a successful relationship.  This activity is divided into 2 parts: in part 1, participants are divided into some small groups (4 to 6 persons). Trainer writes a general topic on the white board (for example going to school in a rainy day) and asks the groups to draw a picture about the topic together in about 10 min, without saying a word. After finishing this stage, all groups should explain others what happens when they should work together without oral conversations. Was it easy or not? Why? What was their alternative ways to solve the challenge? Was it useful enough?  In part 2, the groups are asked to draw another picture, this time following the details that trainer explains to them (trainer explains to them about the picture, only in some parts he/she talks very quietly, so no one can hear correctly). Then groups show their pictures to all and explain why some details are missed or wrong? What is the importance of listening well in communications?  **Final Discussion:** in final discussion, the participants discuss about these questions:   1. What did you learn from the activities today? 2. Was it easy to understand what the session was about? 3. What did you most like/dislike about the session? |


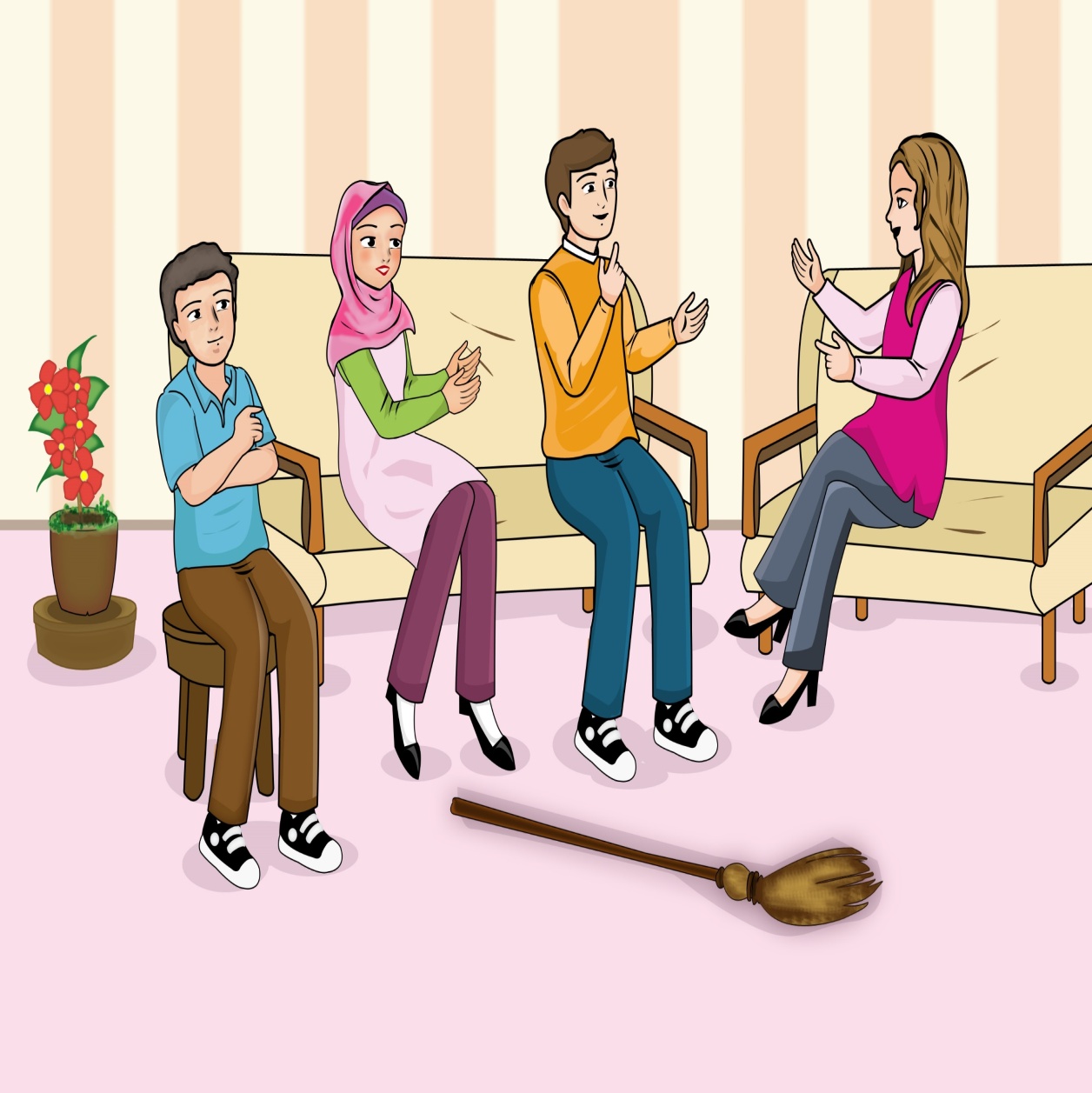


| **Activity 8**  ***I Have the Ability of Effective Negotiation and Saying NO*** |
| --- |
| - **LIFE SKILLES:**   Self-awareness, Communication, Critical Thinking, Critical Thinking, inter- and intra-personal relationships   - **MATERIALS:**   Poster of “Negotiation’s steps”, Colourful pens and pencils, Large blank papers |
| **Purpose, Details and Important Points:**  This activity is purposed to help adolescents to understand the process of a negotiation using six different steps and recognize the different situations in negotiations. Furthermore, this activity helps adolescents to practice the ability to say NO to the dangerous situations during negotiations.  First, adolescents learn about the different steps in negotiation process using the schematic poster of negotiation. Then do practices using short role plays. For example, “your friend always want you to follow them even if you do not agree but this time you want to have a friendly negotiation with him/her to change the situation”. Then trainer explains the situations that adolescents should say a big NO to them instead of any negotiation. For example “when your friend ask you to lie to your parents/caregiver and go to the unknown place with him/her”. Participants then practice some situations through short role-plays.  Then, adolescents learn that in some situations (such as sudden threats) negotiation is not useful and sometimes they should just leave the place or the person behind without wasting time! Participants are asked to explain some situations like that (better if it is their own experience) and discuss about them.  **Final Discussion:** in final discussion, the participants discuss about these questions:   1. What did you learn from the activities today? 2. Was it easy to understand what the session was about? 3. What did you most like/dislike about the session? |


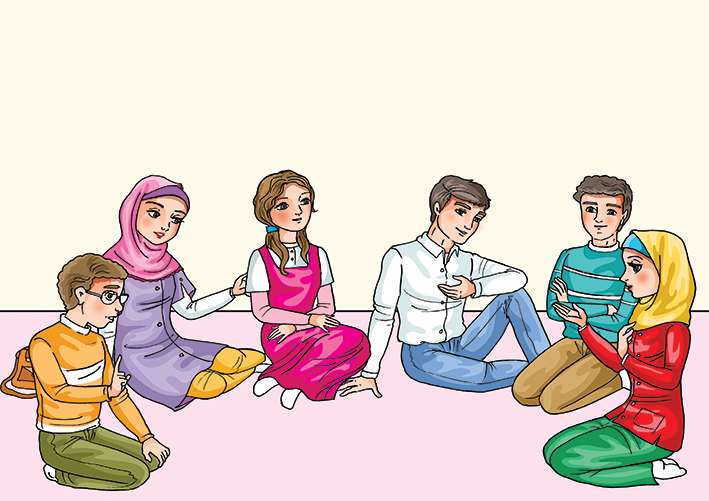

Supplement: S2 File — (DOCX) [file pone.0226333.s002.docx]
